# Octo- and nonagenarians’ outlook on life and death when living with an implantable cardioverter defibrillator: a cross-sectional study

**DOI:** 10.1186/s12877-018-0942-9

**Published:** 2018-10-20

**Authors:** Ingela Thylén, Debra K. Moser, Anna Strömberg

**Affiliations:** 1 Department of Cardiology and Department of Medical and Health Sciences, Division of Nursing Sciences, Linköping University, S-581 83 Linköping, Sweden; 2 College of Nursing, University of Kentucky, Lexington, USA; 3 Sue and Bill Gross School of Nursing, University of California, Irvine, USA

**Keywords:** Attitudes, Deactivation, End-of-life, Implantable cardioverter defibrillator, Knowledge, Nonagenarians, Octogenarians, Psychosocial distress, Quality-of-life, Shock

## Abstract

**Background:**

Elderly individuals are increasingly represented among patients with implantable cardioverter defibrillators (ICD), but data describing life with an ICD are scarse among octo- and nonagenarians. Moreover, few studies have reported those elderly patients’ perspective on timly discussions concerning what shock deactivation involves, preferences on battery replacement, and their attitudes about turning off the ICD nearing end-of-life. Consequently, the aim of the study was to describe outlooks on life and death in octo- and nonagenarian ICD-recipients.

**Methods:**

Participants were identified via the Swedish Pacemaker- and ICD-registry, with 229 octo- and nonagenarians (82.0 ± 2.2 years, 12% female) completing the survey on one occasion. The survey involved questions on health and psychological measures, as well as on experiences, attitudes and knowledge of end-of-life issues in relation to the ICD.

**Results:**

The majority (53%) reported their existing health as being good/very good and rated their health status as 67 ± 18 on the EuroQol Visual Analog Scale. A total of 34% had experienced shock(s), 11% suffered from symptoms of depression, 15% had anxiety, and 26% reported concerns related to their ICD. About one third (34%) had discussed their illness trajectory with their physician, with those octo- and nonagenarians being more decisive about a future deactivation (67% vs. 43%, *p* < .01). A minority (13%) had discussed what turning off shocks would involve with their physician, and just 7% had told their family their wishes about a possible deactivation in the future. The majority desired battery replacement even if they had reached a very advanced age when one was needed (69%), or were seriously ill with a life-threatening disease (55%). When asked about deactivation in an anticipated terminal illness, about one third (34%) stated that they wanted to keep the shocks in the ICD during these circumstances. About one-fourth of the octo- and nonagenarians had insufficient knowledge regarding the ethical aspects, function of the ICD, and practical consequences of withdrawing the ICD treatment in the end-of-life.

**Conclusions:**

Increasing numbers of elderly persons receive an ICD and geriatric care must involve assessments of life expectancy as well as the patient’s knowledge and attitudes in relation to generator changes and deactivation.

## Background

For over 20 years, implantable cardioverter defibrillators (ICDs) have been the treatment of choice for individuals at risk for - or with a history of - sudden cardiac death attributable to ventricular arrhythmias [1]. When ICDs are combined with cardiac resynchronisation therapy (CRT) in heart failure patients, symptom burden and survival are improved [1]. Therefore today, elderly individuals are increasingly represented among patients with ICDs and studies have shown that > 40% of ICDs and CRT devices are inserted in individuals ≥ 70 years of age [2]. About 12% of those deemed eligible for ICD implantation by conventional criteria are ≥ 80 years of age (i.e., octogenarians) [2]. Although guidelines state that ICD implantation is “rarely appropriate” in nonagenarians (i.e., ≥ 90 years of age) [3], they do not refer to any specific age limits, but rather to 1-year life expectancy as a mandatory criterion.

As the population ages, many are at greater risk of developing progressive multimorbidity, and therefore are likely to die of causes other than sudden cardiac death [4]. Under these circumstances, shocks may only cause a prolongation of the dying process [5]. During the first years after implantation, the risks of receiving at least one ICD shock can range from one third in primary preventive patients [6, 7] to up to 50% in patients with a secondary indication [8]. Even among those without shocks during the ICD’s first battery life, the incidence of shocks at 5 years following generator exchange is > 25% [6] which can negatively affect quality-of-life (QOL) [9]. A recent systematic review that assessed > 5000 ICD-recipients reported that approximately 20% have clinically significant psychological distress with anxiety and/or depression the first year(s) post-implantation [10]. Furthermore, 31% receive a shock(s) in the last 24 h of their lives, decreasing their quality-of-death [5].

An ICD deactivation should be considered when a patient’s clinical status worsens and death is near. Deactivating an ICD can be achieved in the following ways, which are ethically and legally equivalent: planned ICD deactivation through reprogramming, urgent temporary ICD deactivation using a magnet which temporarily stops the ICD delivering shocks whilst in position over the ICD, or not replacing the ICD when the device has reached the end-of-service indicator [11].

Effective communication between clinician and patient includes determining the patients’ goals of care, helping patients to weigh the benefits and burdens of device therapy as their clinical situation changes, clarifying the consequences of deactivation, and discussing potential alternative treatments [12]. Therefore, in order to improve QOL for patients at the end of their lives, and to provide direction for clinicians, it is adviced that clinicians raise the question about elective ICD deactivation before implantation of the device and then recurrently during the illness trajectory [11, 13], yet few patients or families discuss the option of device deactivation with their physicians [14]. Many do not know that device deactivation is an available option, [15] which may result in a potentially painful end-of-life situation with the ICD not being deactivated [16]. There are several obstacles to effective communication and patient education regarding this issue. Firstly, many professionals still feel uncomfortable discussing end-of-life with their patients [17] and lack the competence and skills to communicate about end-of life issues [18, 19]. Secondly, professionals may feel uncertain about if and/or when patients prefer to discuss generator replacement and deactivation, which could result in a delayed end-of-life discussion [20]. Thirdly, patients might not want to discuss this topic at all [14, 21, 22].

Despite the increasingly high rate of ICD implantation in elderly patients, data describing their life-situation, experiences and perspectives on the timing of ICD deactivation discussions, and preferences on deactivation as the patient nears end-of-life, are scarse. Therefore, the overall purpose of this study was to describe outlook on life and death in octo- and nonagenarian ICD-recipients. The specific aims are as follows, using a cross-sectional design: 1) to describe self-rated perceived health and psychological distress; and 2) to describe experiences, attitudes and knowledge of end-of-life issues.

## Methods

In 2012, we used the Swedish ICD- and Pacemaker registry to identify and invite all eligible ICD- and CRT-D (cardiac resynchronization therapy with defibrillator) recipients (*n* = 5535) at the time to complete standardized self-reported questionnaires, on one occasion. The survey involved questions on health and psychological measures, as well as on experiences, attitudes and knowledge of end-of-life issues in relation the the ICD/CRT-D. The inclusion criteria were being eligible for the registry (i.e., having a valid postal mail address), being ≥ 18 years old, having had the device for at least 1 year, and willingness to participate. The study was approved by the Regional Ethics Committee for Human Research at the University of Linköping, Sweden (Dnr 2011/434–31). The results from the main study are reported elsewhere [14, 23–25]. In this sub-study we have analysed data from those ≥ 80 years old.

### Measures

#### Demographic and clinical data

Demographic data, co-morbidity and previous shock experience were obtained through purpose-designed questions whereas clinical variables such as primary vs. secondary indication and time since implantation were obtained from the Swedish ICD- and Pacemaker registry.

#### Quality-of-life and psychological measures

Quality-of-life was measured using the *EuroQol-5D* (EQ-5D) [26], an instrument with well-established reliability and validity. Symptoms of anxiety and depression was measured using the *Hospital Anxiety and Depression Scale* (HADS) [27], which has been used extensively in both hospitalized and non-hospitalized patients. The literaure supports the use of ≥8 as a cut-off, as it yields an optimal balance between sensitivity and specificity for the presence of symptoms of anxiety and depressive symptoms [28]. *ICD-related concerns* was measured using the 8-item Patient ICD Concerns (ICDC) questionnaire, which is a brief instrument that can be used to identify patients at risk for adverse outcomes in clinical practice. [29]. As there is no standardized cut-off for the ICDC, the instrument constructors [30] suggest dividing patients into a high vs. low concern group, using the highest tertile in the sample to indicate a high level of concerns. Perceived social support from family, friends, and significant others was measured using the *Multidimensional Scale of Perceived Social Support* (MSPSS) [31]. The higher total score, the higher the perceived social support. In addition, separate subscales can be used by summing the responses from the items in each of the three dimensions. The 4-item, *Control Attitudes Scale* (CAS) was used to measure perceived control [32]. Higher scores indicate greater perceptions of control. As recommended from the literature, we used the median in the sample as a cut-off for low vs. high perceived control [33].

#### End-of-life concerns

Data on experiences, attitudes and knowledge of end-of-life concerns was measured using the *EOL-ICD Questionnaire* [34]. The EOL-ICDQ is a self-rated questionnaire containing three domains that measure experiences (10 items), attitudes (18 items) and knowledge (11 items) of end-of-life in ICD-recipients. Participants list their answer as “yes/no” or “no opinion”, “agree/don’t agree”, “true/false”, or “don’t know”. The experiences domain includes items about patients’ actual discussion experiences. Example items in this domain are “I have discussed what a battery replacement involves with my ICD doctor or nurse”, and “I have told my next of kin (either in writing or orally) my wishes regarding the shocks in my ICD, if I become seriously ill with some fatal disease”. The attitudes domain includes items about patients’ attitudes towards potential future discussions and future events. Examples of items are “I want to have the battery in my ICD replaced even if I am seriously ill suffering from another disease” and “I want to have the shocks in my ICD even if dying of cancer or another serious disease”. The knowledge domain involves 3 statements on ethical aspects, 2 statements on the fuction of the ICD, and 6 statements on practical consequences associated with ICD deactivation, such as “Deactivating the shocks is the same as active euthanasia” (i.e., false) and “When the shocks are turned off, the heart stops beating” (i.e., false).

### Statistical analysis

All analyses were conducted using SPSS software, version 22 (SPSS, Chicago, Illinois). We used frequencies and proportions to describe the sample, their self-rated perceived health and psychological distress, as well as their experiences, attitudes and knowledge of end-of-life issues. Mann-Withney U-tests and chi-square tests were used to determine bivariate associations of socio-demographic, clinical, and psychological measures with positive and negative outlook on life and death (i.e., self-rated perceived health, willingness to discuss deactivation, attitudes on elective generator replacement at end-of-service indicator, and attitudes about maintaining ICD therapy in the context of terminal illness). As recommended in the literature [16], the 25th percentile in the sample (i.e., ≤3) was used as a cut-off for insufficient knowledge in the knowledge domain in the EOL-ICDQ. Probability values of *p* < .05 were considered significant.

## Results

### Background characteristics

Of the 5535 patients who were mailed the survey, 12% were > 80 years of age. A total of 3067 patients completed the survey, covering 55% of all Swedish ICD-recipients at the time for the study. Of these, 229 (7,5%) were octo- and nonagenarians and included in this analysis. The mean age of this sub-sample was 82.0 ± 2.2 years with a range of 80 to 94 years, with the majority being male (88%). Time since implantation ranged from one to 23 years with a mean of 5.5 ± 4.2 years, 25% had a CRT-D implanted and 32% had previously undergone an elective battery replacement. The majority (72%) had received their ICD as secondary prevention treatment, while the remainder had received their ICD for primary prevention, usually in the context of heart failure. The number of co-morbid conditions in total varied from 0 to 11, with 57% of the patients having ≥3 co-morbid conditions (mean 3.1 ± 1.9) (Table 1).

**Table 1:** Background characteristics, *N* = 229

Characteristic^a^	Value^b^
*Demographics*	
Age (years)	82.0 (2.2)
Gender (male)	201 (87.8%)
Education (lower)^c^	102 (44.5%)
Living alone (yes)	55 (24.3%)
*Clinical factors*	
Time since implantation (years)	5.5 (4.2%)
ICD-indication (primary prevention)	65 (28.4%)
Resynchronization therapy (CRT-D, yes)	57 (24.9%)
Shock experience (yes)	76 (33.9%)
Generator replacement (yes)	73 (31.9%)
*Co-morbidity*	
Myocardial infarction	92 (40.2%)
Atrial fibrillation	125 (54.6%)
Heart failure	131 (57.2%)
Chronic obstructive pulmonary disease	32 (14.0%)
Diabetes mellitus	33 (14.4%)
Stroke	27 (11.8%)
Cancer	29 (12.7%)

^a^Self-reported by subjects

^b^Data are presented as mean (SD) or n (%)

^c^Compulsory secondary school, with a total education time ≤ 9 years

### Outlook on life

**Fig. 1:**
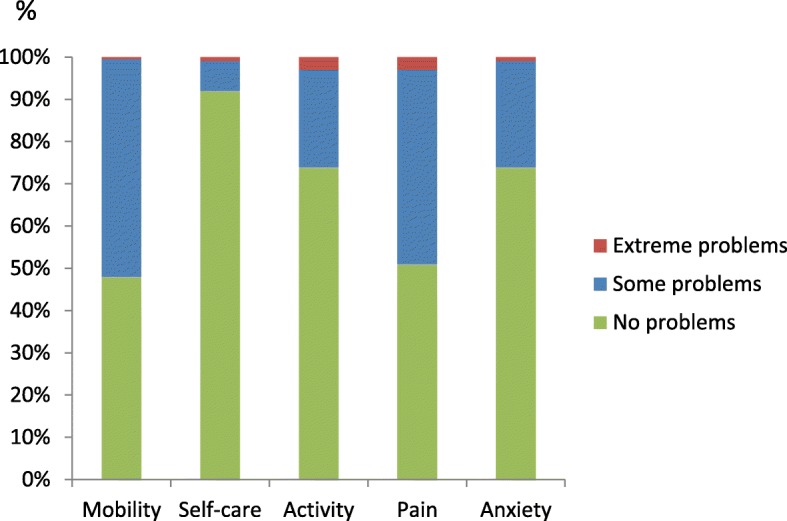
ICD-recipients perceived health, measured with EQ-5D

The vast majority rated their general experiences as ICD-recipients as “good” or “very good” (97%). The corresponding percentage for their perceived general health experience was 53%, with a EuroQol VAS score ranging from 10 to 100 (67 ± 18) and a mean index of 0.783. The lowest proportion of reported health problems was seen in the self-care dimension (8%) whereas the highest proportion of problems were seen in the mobility dimension (52%) (Fig. 1). A worse health condition (i.e., EQ-VAS) was described by patients with a history of myocardial infarction (64 vs. 70, *p* < .05), heart failure (64 vs. 71, *p* < .01), kidney disease (57 vs. 69, *p* < .01), or intermittent claudiocation (60 vs. 69, *p* < .001) compared to their counterparts. Also patients with symptoms of depression and anxiety, and ICD-related concerns reported a worse health condition (EQ-VAS 44 vs. 70, 47 vs. 71 and 61 vs. 70, respectively, *p* < .000).

In total, 11% reported symptoms of depression, 15% anxiety, 26% had ICD-related concerns, and 32% described a low perceived control in life (Table 2). Those with symptoms of anxiety also perceived a lower control compared to them without anxiety (52% vs. 28%, respectively, *p* < .01). This was also true for the patients with high ICD-related concerns; 45% described a low control in this group compared to 26% in the group without any concerns (*p* < .01).

**Table 2:** Psychological characteristics, N = 229

Characteristic^a^	Value^b^
*Perceived ICD experience*	
Experiences of the ICD-treatment (“good/very good”)	213 (96.8%)
Pain experience with the latest shock, numeric rating scale (score 0–10)	4.1 (3.2)
Anxiety experience with the latest shock, numeric rating scale (score 0–10)	3.6 (2.9)
*Quality-of-life (EQ-5D)*	
Quality-of-life index, mean	783 (.212)
Quality-of-life, visual analogue scale, total (score 0–100)	67.4 (18.4)
Poor quality-of-life, visual analogue scale, 0–65	71 (34.1%)
Moderate quality-of-life, visual analogue scale, 66–79	74 (35.6%)
Good quality-of-life, visual analogue scale, 80–100	63 (30.3%)
*Psychological distress*	
Symptoms of anxiety (HADS-A)	32 (14.6%)
Symptoms of depression (HADS-D)	25 (11.2%)
ICD-related concerns (ICDC)	57 (26.1%)
Low perceived control (CAS)	71 (32.0%)
Percieved social support (MSPSS), (score 12–84)	71.4 (15.1)

^a^Self-reported by subjects

^b^Data are presented as mean (SD) or *n* (%)

**Fig. 2:**
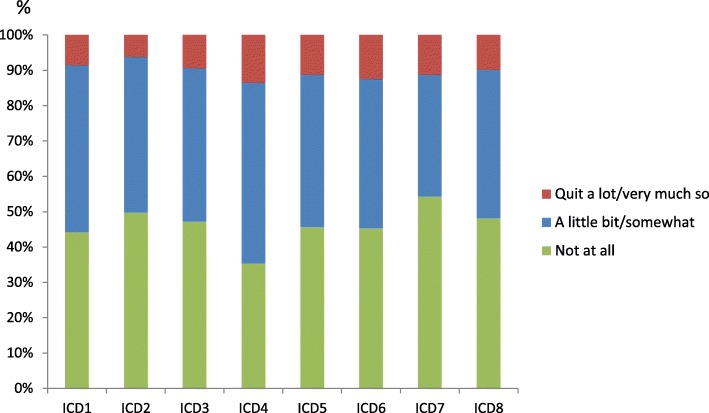
ICD-related concerns, measured with ICD-C. 1) My ICD firing; 2) Doing activities/hobbies that may cause my ICD to fire; 3) Time spent thinking about my ICD firing; 4) Working too hard/overdoing things causing my ICD to fire; 5) Having no warning my ICD will fire; 6) The symptoms/pain associated with my ICD firing; 7) Not being able to prevent my ICD from firing; 8) Getting too stressed in case my ICD fires

About one third of the patients (34%) had received at least one shock and that experience was also correlated with a higher percentage of symptoms of anxiety compared to those without any shock experience in the past (24% vs. 10%, respectively, *p* < .01). In connection with the latest shock, they rated their pain and their anxiety experience as 4.1 and 3.6 on a 10 point numeric rating scale, respectively. The ICD-recipients were least concerned about not being able to prevent the ICD from firing, with 46% being concerned for this reason. ICD-recipients were most concerned that working too hard/overdoing things might cause the ICD to fire, with 65% sharing this concern (Fig. 2). A minority (27%) stated that they had a religious faith or outlook on life helping them in their daily life as an ICD-recipient.

### Outlook on death

**Fig. 3:**
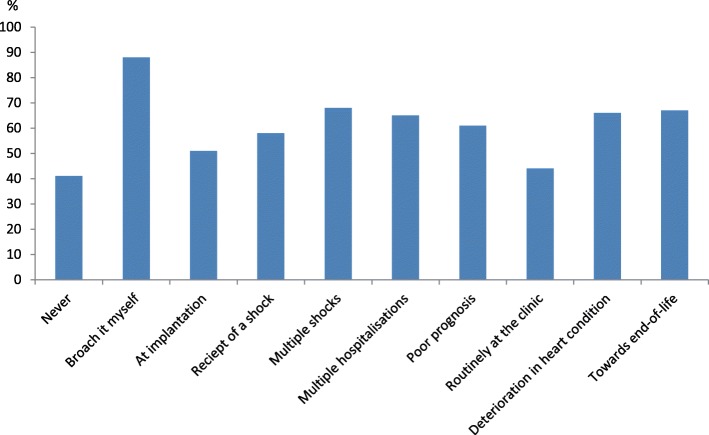
Octo-nonagenarians preferences about the timing to discuss what deactivation involves

Nearly one forth (24%) of the octo- and nonagenarians stated that they often thought about death, and one third (34%) had discussed their illness trajectory with their physician. About 40% had at some time discussed what a forthcoming generator replacement would involve for them, and 30% had discussed the topic with a family member. The vast majority (87%) had not discussed with their physician about what turning off the shocks would involve if their health deteriorates, and just 7% had told their family their stance about deactivation when nearing end-of-life. Among those who had discussed their wishes, 93% perceived a high control in life compared to 65% among those that had not discussed this issue with their family (*p* < .05). Six participants had considered deactivation in the future. Many of the participants (40%) stated that they under no circumstances wanted to discuss deactivation. Among those who wanted to discuss deactivation, 51% found it appropriate to have the discussion at implantation, while 61% wanted to discuss deactivation if suffering from a poor prognosis, and 67% towards the end-of-life (Fig. 3).

**Fig. 4:**
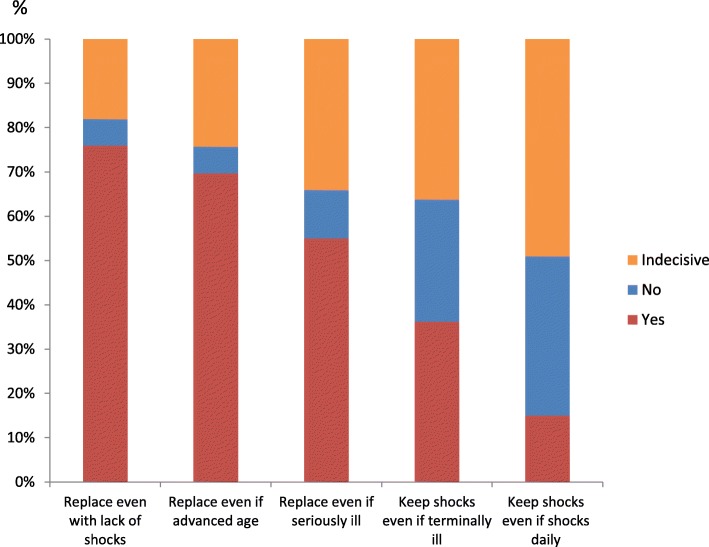
Octo-nonagenarians preferences about if to replace the ICD battery when it has reached the end-of-service indicator given three different scenarios, and if keep shocks in an anticipated future with worsening of the health

When being faced with three different scenarios at the time for the end-of-service indicator in the ICD, most of the participants stated that they would like to replace the ICD even if no shock therapy had been delivered (76%), if had reached a very advanced age (69%), or if being seriously ill in a life-threatening disease at that time (55%). When asked about deactivation in an anticipated terminal illness, about one third of patients (34%) stated that they wanted to keep the shocks in the ICD during these circumstances, and 15% wanted to keep the shocks even if having shocks daily (Fig. 4). Those with a CRT-D implanted were more able to take a stand about deactivation (yes or no) if falling ill in a terminal illness compared to those without a CRT-D (77% vs. 62%, respectively, *p* < .05). This was also true for participants with a history of myocardial infarction when compared to their counterparts (76% vs. 60%, respectively, *p* < .01). Furthermore, those who had discussed deactivation with their family were more able to take a stand about deactivation in an anticipated terminal illness (86% vs. 65%, respectively, *p* < .05). When it came to an anticipated situation with daily shocks, again those with a history of myocardial infarction were more able to take a stand (yes or no) about deactivation compared to those without myocardial infarction (60% vs. 45%, respectively, *p* < .05). Those who hade discussed their illness trajectory with their physician were also more decisive about this situation with 67% being able to take a stand about deactivation compared to 43% among those who had not discussed their illness trajectory with their physician, *p* < .01. Finally, those who were willing to discuss deactivation with their physician had less often had a battery replacement (44% vs. 66%, *p <* .01), more often a history of atrial fibrillation (68% vs. 49%, *p* < .01) or diabetes (75% vs. 57%, *p* < .05). There were no other significant differences in the bivariate analyses between different socio-demographic, clinical, or psychological measures and those who were willing to discuss deactivation of the ICD, or were able to take a stand about deactivation (yes or no) or were indecisive about their standpoint in case of terminal illness or having shocks daily.

Only two participants (1%) recieved the highest possible points on the knowledge questionnaire, whereas 17 participants (8%) did not know or scored incorrectly on all statements. The mean score in the sample was 5.6 ± 3.0 out of 11 possible scores, with 54 participants (26%) having insufficient knowledge. In the ethical domain, the statement “When an ICD’s defibrillating shocks are turned off, the heart stops beating,” (i.e., false) resulted in the highest score with 74% responding correctly. Approximately one third (36%) in-correctly believed that deactivation was the same as active euthanasia. The two statements in the domain covering the function of the ICD, i.e., “When the battery voltage is beginning to wear, the ICD’s functioning worsens” and “When the shocks are turned off, the pacemaker function in the ICD is also turned off,” were correctly responded to as false by 23% and 30%, respectively. In the last domain about practical consequences of drawing the ICD treatment at the end-of-life, only 24% scored correct (i.e., false) to the statement “An ICD always delivers defibrillating shocks in connection with end-of-life”. Additionally, approximately one third of the octo- and nonagenarians (37%) incorrectly believed that the ICD must be removed by surgery in order to be turned off, and 44% believed that when the ICD has been deactivated, it cannot be activated again.

## Discussion

Our study, conducted in a national sample of octo- and nonagenarian ICD-recipients, demonstrated that nearly one-third of the participants experienced a good QOL and their overall ICD experience was considered good or very good in almost all the participants. Further, they reported similar prevalence of symptoms of anxiety and depression as in a Swedish norm population in this age group [35]. As many as 26% had ICD-related concerns and were most concerned about that working too hard/overdoing things may cause the ICD to fire. This is noteworthy since fear may influence and decrease activity levels in daily life [36, 37] which in turn has been found to strongly correlate with survival following ICD-implantation [38].

We found that only a minority had discussed the implications of generator replacement at battery-end-of-service or had discussed potential ICD deactivation at the end-of-life. Moreover, many participants were unable to foresee what they might prefer to do with their ICD in an end-of-life situation. Two thirds of the participants in our study wanted to replace their battery irrespectable of age and half of them wanted this done also when being seriously ill. As many as one third required to keep the ICD active until they died. These findings suggest that many patients with an ICD are at risk of not dying peacefully, painlessly and with dignity. It is the reality that many dying ICD-recipients receive shocks in their last days of life. A recent Swedish study by Westerdahl et al. showed that one out of four patients received at least one shock during the last day of their life. It is noteworthy that this study also showed that ICD devices were active in half of patients with a do-not-resuscitate order [19].

Earlier studies have shown that patients and their family members - as well as healthcare professionals - are reluctant to speak about not replacing the battery or deactivation [17, 21, 39]. Our study underlines these findings and showed that remarkably few had discussed deactivation with their physician and/or family members. A study by van der Wal et al. found that many elderly and severly ill cardiac patients do not know what to expect for the future and some have unrealistic exectations on their long-term survival [40]. Older people obviously have a higher annual rate of death – with a 38% mortality rate 2 years after ICD replacement in octogenarians reported in a recent study [41], and this fact must be taken into consideration when discussing future expectations with the patient. Our study further showed that about one-forth of the participants had insufficient knowledge regarding the ethical aspects, function of the ICD, and practical consequences of withdrawing the ICD treatment in the end-of-life. We have previously shown that increased knowledge improve the compentences and skills of making a decision, in favour of deactivation or not [16]. Dodson et al., [42] found that providing information regarding the best current evidence on benefits and burdens of an ICD with focus on person-centered outcomes, triggered that ICD-recipients made a decision for deactivation in at least 1 out of 5 end-of-life scenarios [42]. However, the lack of knowledge is not just on the patient side. Many physicians, medically responsible for elderly patients with an ICD, have limited knowledge of ICD treatment and may therefore not be able to provide the best possible care from implantation to the end-of-life in the elderly ICD-recipients [19]. Thus, we see a need for more education on ICD care and communication about deactivation to physicians both in geriatrics, internal medicine and primary care as well as a more close collaboration with cardiologists and device nurses responsible for ICD follow-up.

### Methodological issues

The study has several limitations with the design being cross sectional and data collected by self-report. Only one data collection point was used, therefore it is not known if the individual’s psychological distress existed before the ICD-implantation. A longitudinal study of a large cohort of older ICD-recipients would be helpful in understanding the process of adjustment, changes in psychological distress and outlook on death over time. Even thought this is a national registry based study, we may have had a selection bias with those octo- and nonagenarian ICD-recipients responding to the survey being less burdened by psychosocial distress and having better QOL. However, a reasonable response rate and a large sample size contributed strength to the results. Lastly, more than 60% of participants had ICD implanted as secondary prevention. This is a relatively high rate for current practice which could be seen as a limitation, but could be explained by the rather long time since implantation ranging up to 23 years.

## Conclusions

We found that most octo- and nonagenarian ICD-recipients report a good health with similar prevalence of psychological distress as younger ICD-recipients and other populations in the same age group. They view their ICD experience as positive and continue to desire ICD therapy in spite of age or serious illness. However, a significant majority of patients have not discussed possible future deactivation with their physician or family. Misunderstandings regarding withdrawing the ICD treatment in the end-of-life were evident, which may result in a potentially painful end-of-death with the ICD not being deactivated. Increasing numbers of elderly persons receive an ICD and geriatric care must remember involving assessments of life expectancy as well as the patients’ knowledge and attitudes in relation to generator changes and deactivation. These conversations should continue over the course of the patient’s lifespan, since patient preferences may change as illness progresses and deactivation needs to be a deliberate decision based on knowledge.

## Abbreviations

CAS: The 4-item Control Attitudes Scale
CRT: Cardiac resynchronisation therapy
CRT-D: Cardiac resynchronisation therapy with defibrillator
EOL-ICDQ: The Experiences, Attitudes and Knowledge of End-of-Life Issues in Implantable Cardioverter Defibrillator Patients Questionnaire
EQ-5D: EuroQol-5 Dimensions
EQ-VAS: EQ-5D visual analogue scale
HADS: The Hospital Anxiety and Depression Scale
ICD: Implantable cardioverter defibrillator
ICDC: The 8-item Patient ICD Concerns questionnaire
MSPSS: The Multidimensional Scale of Perceived Social Support
QOL: Quality-of-life
